# A chemically-aware validation framework for benchmarking large language models in materials synthesis planning

**DOI:** 10.1186/s13321-026-01222-5

**Published:** 2026-05-24

**Authors:** Aobo Zhang

**Affiliations:** Department of Chemistry, Tsinghua University, Beijing, 100084 China

**Keywords:** LLM benchmarking, Evaluation metrics, Generative AI, Scientific NLP, Automated synthesis, Cheminformatics

## Abstract

**Abstract:**

We present a domain-tailored verification framework for evaluating the scientific quality of AI-generated synthesis protocols, moving beyond generic NLP benchmarks that fail to capture chemistry-specific requirements. Our approach combines two quantitative metrics: a framework score that assesses the logical coherence of the synthesis pathway, and a weighted detail score that measures the precision of reported experimental parameters.

**Scientific Contribution:**

This work establishes a benchmark for automated protocol generation, quantifies the gap between conceptual feasibility and parametric exactness in LLM outputs. We apply carefully curated dataset of SAC as a testbed to fine tune mainstream open source LLMs. The benchmark can be generalized to material synthesis protocols.

**Supplementary Information:**

The online version contains supplementary material available at 10.1186/s13321-026-01222-5.

## Introduction

Self-driving laboratories (SDLs), structured around Design-Build-Test-Learn (DBTL) cycles, promise to accelerate materials discovery by automating synthesis and optimization [1, 2]. Large language models (LLMs) have recently emerged as powerful tools for the “Design” stage, capable of extracting and generating synthesis procedures from the scientific literature [3]. Yet their probabilistic nature introduces a validation gap: syntactically correct instructions can be chemically implausible or parametrically infeasible, leading to costly failed experiments [4, 5]. Robust, chemistry-aware evaluation frameworks are therefore essential to ensure the trustworthiness of AI-driven design before integration into SDLs.

Addressing this challenge requires a fundamental rethinking to quantitatively evaluate the performance of LLMs. As shown in Table 1, recent related works have not studied the assessment of material synthesis plan generated by LLMs. In addition, current natural language processing (NLP) benchmarks such as BLEU or ROUGE are designed to measure surface-level textual similarity, not the validity of scientific reasoning [6, 7]. Conventional NLP metrics fail to capture whether a generated synthesis plan adheres to mechanistic logic, balances stoichiometry, or specifies parameters within feasible ranges. Without evaluation frameworks that directly interrogate domain-grounded correctness, progress in applying LLMs to science risks being misled by metrics divorced from practical relevance. Indeed, as we will demonstrate, reliance on conventional NLP metrics can be actively misleading, prioritizing linguistic mimicry over the scientific reasoning essential for experimental success. We quantitatively grounding this critique for the first time in continuous synthesis steps involving complex operational parameters.

**Table 1:** Comparison of recent high impact LLMs in chemistry

Model	Assessment	Field
This work	Dual layer expert assessment (framework and detail)	Single-atom catalysis (SAC)
Chemma [8]	Top-k precision	Organic chemistry
L2M3 [9]	Machine learning simulation	Metal organic framework

Here, we propose a two-tiered quantitative evaluation framework to close this verification gap. The framework consists of a "framework score", which assesses whether an LLM-generated synthesis strategy is chemically coherent at a structural level, and a "detail score", a weighted measure that evaluates the accuracy of critical synthesis parameters. To rigorously test this approach, we fine-tuned LLMs based on knowledge of the synthesis of single-atom catalysts (SACs), which is an ideal testbed due to its high dimensionality of choices, extreme sensitivity to synthesis conditions, and zero tolerance for forming nanoparticles [10, 11]. In SAC synthesis, an incorrect precursor, solvent, or thermal treatment step invalidates the entire process, making it a stringent setting in which to probe the robustness of AI-driven scientific design [12, 13]. This application context therefore serves not as an end in itself, but as a proving ground to demonstrate the necessity and effectiveness of the proposed evaluation methodology.

Building upon the long-standing tradition in cheminformatics of using computational methods to evaluate and guide chemical discovery, this work extends the scope of model assessment into a new frontier. Previous studies have, for example, focused on evaluating the robustness of molecular property prediction models or developing novel algorithms for molecular generation [14–17]. However, a clear validation gap remains when it comes to assessing natural language synthesis protocols produced by large language models, protocols that encapsulate full procedural steps rather than isolated chemical descriptors. Our study aims to address this emerging challenge directly by establishing a systematic framework for verifying the scientific reliability of AI-generated synthesis procedures.

In summary, our contributions are threefold: First, we introduce a novel and quantitative validation framework for assessing the reliability of AI-generated scientific processes. Second, we demonstrate that in specialized domains, carefully fine-tuned open-source LLMs can outperform larger, general-purpose commercial LLMs. Last but not least, we construct and validate the core "cognitive module" necessary for future autonomous scientific discovery agents.

## Methods

**Fig. 1:**
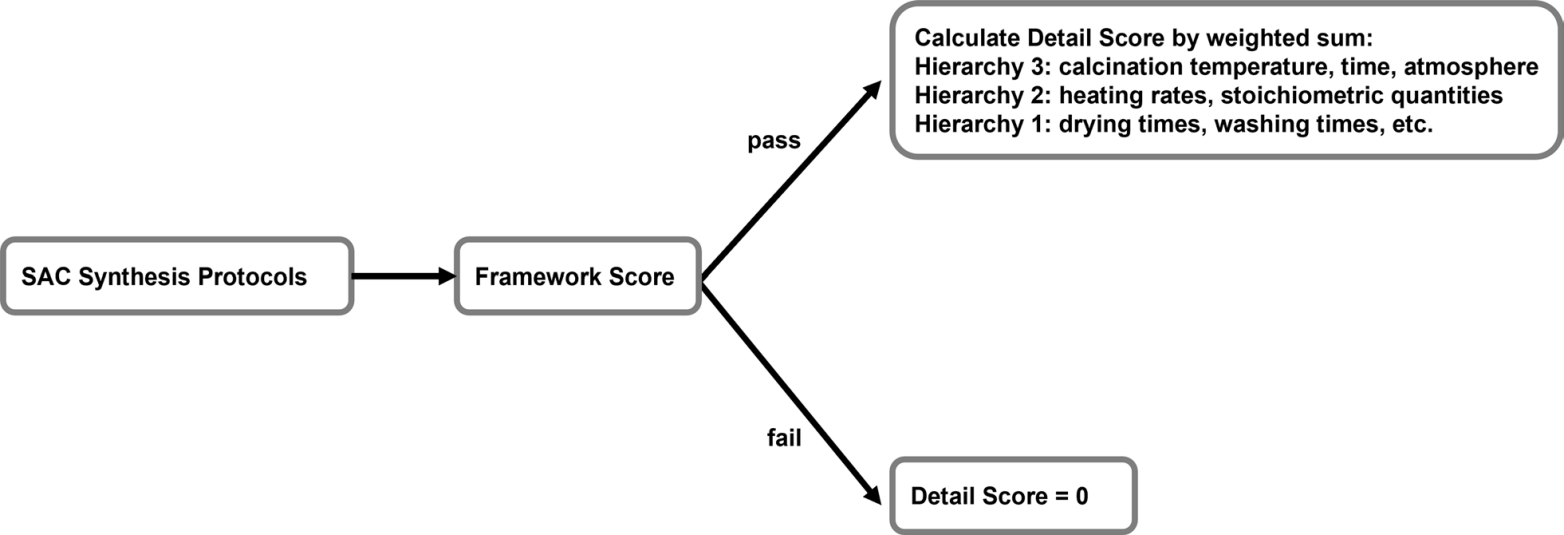
Workflow of this quantitative evaluation system

The quantitative and expert-driven evaluation framework is composed of a framework score and a detail score, as shown in Fig. 1. The overall framework of synthesis design is judged with a value “pass” (1) or “fail” (0), defined as framework score. The framework score serves as a critical initial filter for chemical plausibility against hallucinations. A generated synthesis protocol must first pass this binary check, which ensures correct precursors, support, and sequence of major steps (e.g., impregnation, calcination), before a more granular analysis of its quantitative details. This prevents meaningless further evaluation of nonsensical or fundamentally flawed procedures.

If the synthesis design receives a “pass” in framework score, it is then count on the key details it hits such as the amount of each chemical and the temperature of calcination. This score is defined as detail score and is normalized to unity. The detail score is set to 0 if the synthesis design receives a “fail” of framework score. The hierarchy for the detail score is designed to reflect the hierarchical impact of parameters on the final SAC properties, as shown in Table 2. Calcination conditions (temperature, time, atmosphere) are assigned the highest level (3) as they directly govern the formation, coordination, and stability of the single-atom sites, which are the heart of SAC. In this high-temperature process, the decomposition of a precursor material traps individual metal atoms within a carbon matrix to form SAC. Stoichiometric quantities and heating rates are assigned a moderate level (2), as they are critical for precursor conversion and support morphology but allow for some procedural latitude. Other procedural parameters (e.g., washing, drying times) receive the lowest level (1) as they are important for reproducibility but typically have a secondary effect on the catalytically active sites themselves.

**Table 2:** Classification of weights for detail score

Parameter genre	Examples	Level	Explanation
Thermal conditions	Temperature, heating time, atmosphere	3	Core of SAC synthesis, determine active sites
Stoichiometric quantities, heat rate	Precursor amount, support amount, solvent volume	2	Important but allow small imprecision
procedural parameters	Drying time, washing time	1	

The hierarchical structure is not arbitrarily assigned but rather reflects the widely recognized parameter sensitivities in the field of SAC synthesis. For instance, thermal treatment conditions (calcination) are assigned the highest hierarchy as they are paramount in controlling single-atom anchoring, preventing aggregation via sintering, and ultimately defining the active site's coordination environment and stability [18–20]. We introduce a multidimensional sensitivity analysis to demonstrate the robustness and consistency of the evaluation framework under varying weight preferences, thereby establishing its reliability as a standardized benchmark. We quantify this hierarchy by assigning different weight combinations for this fixed hierarchy level. For example, the three levels are assigned weights combination 3-2-1 (level 3 is assigned weight 3 and so on) as a starting point. Furthermore, to eliminate subjective bias of weight assignments, we also evaluated alternative weight combinations 10-2-1 and 1-1-1 for comparison.

The total weight was the sum of all weights from all detail factors. The score of each detail was calculated depending on its type. If the detail was non-numerical, then its score was its weight fraction if the generated value matched, or 0 if the generated value was missing or not matched. If the detail was numerical, its score was calculated by the following equation:

$${score}_{i}= \frac{{w}_{i}}{\sum {w}_{i}} (1 - \frac{|{v}_{generated}-{v}_{literature}|}{{v}_{literature}})$$

where w_i_ is the weight of key detail i determined by above 3 level of importance, v_generated_ is the value generated by LLMs and v_literature_ is the value provided by literature. In case the value generated was more than 100% off from the value from literature, the detail score may become negative. Then the detail score was taken as 0 if it became negative.

To test the effectiveness of this benchmark, we apply this evaluation system to the generated SAC synthesis protocols by fine-tuned LLMs. We chose QLoRA as a practical and efficient approach given the relatively small dataset size. By freezing most pretrained weights, QLoRA effectively preserves this valuable general reasoning capability, while its low-rank adapters efficiently inject domain-specific chemical knowledge in SAC [21, 22].

The open-source pre-trained LLMs selected for fine-tuning by QLoRA include Qwen1.5-7B, Baichuan2-7B-Base and Llama-3-8B. We choose these LLMs because they show exceptional performance especially in reasoning, and meanwhile they are of similar weight size, suitable of our computational resource costs but strong enough for our tasks. Multiple LLMs are chosen for comparison to find the potentially most appropriate for reasoning of SAC synthesis. The commercial model chosen without our fine-tuning is Deepseek-R1-0528 for comparison. We choose Deepseek-R1-0528 because it, as an open-source LLM, shows outstanding reasoning performance close to current world leading models such as OpenAI-o3 and Gemini-2.5-Pro [23].

**Fig. 2:**
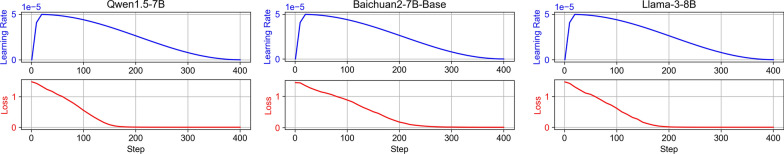
Monitored learning rates and losses of fine-tuning for selected LLMs

All the selected LLMs for fine-tuning are with the same hyperparameters including epoch of 400, batch-size of 50, tensor rank of 64, etc. The hyperparameter configuration is engineered for a scenario demanding deep and stable adaptation of a pre-trained LLM to a specialized task. The moderate epoch count ensures comprehensive learning and meanwhile avoids overfitting; the moderate batch size provides a regularizing yet efficient optimization signal; and the substantial tensor rank grants the model ample, but not excessive, capacity for change. This triad works in concert to facilitate a training process that is both effective in its outcome and efficient in its implementation. The same prompt template and system template are applied for all LLMs including commercial LLM such as Deepseek-R1-0528 to ensure the fairness of performance comparison. Although independent hyperparameter optimization for each model might yield their respective optimal performances, we adopted a fixed set of hyperparameters to ensure that the observed performance differences primarily stem from the models’ underlying architectures and pretraining processes, which constitute the central focus of our comparative analysis. The loss and learning rate are monitored during fine-tuning for all LLMs to avoid overfitting and gradient explosion, as shown in Fig. 2. The convergences of losses and the smooth drops of learning rates confirm the proper hyperparameters selected for fine-tuning.

**Fig. 3:**
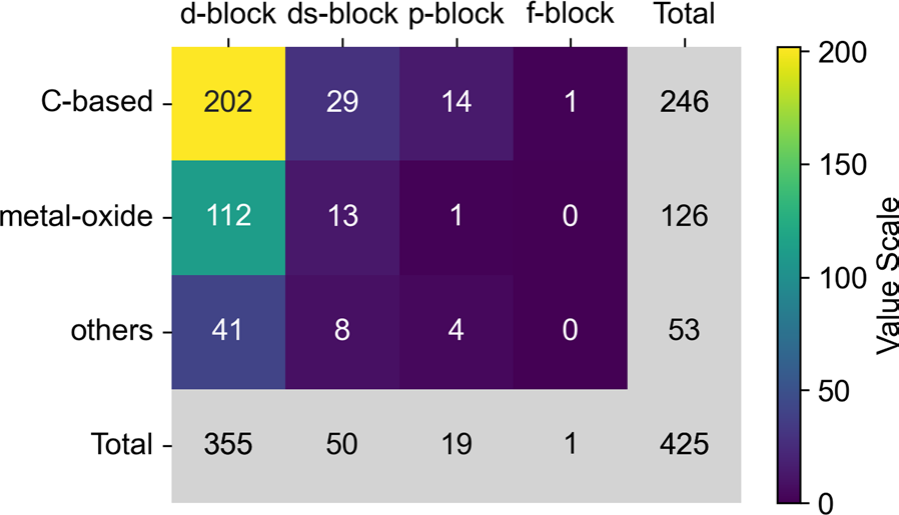
Classification of collected synthesis protocols

To train robust LLMs, we compile a dataset of 425 SAC synthesis protocols in 239 papers from high-impact journals, including Nature, Nature Chemistry, Nature Materials, Journal of the American Chemical Society, and related publications. The selection of papers is determined by the impact of journals, the number of other papers on similar topics, the difficulties to validate the experimental procedures, and the clarity of the synthesis process narration. To ensure data quality and reproducibility, protocols are curated exclusively from peer-reviewed articles in leading chemistry journals (Impact Factor > 15). Protocols are selected if they were reported by at least three independent research groups to ensure a high degree of consensus and reproducibility. Protocols with more than one intermediate product are omitted since these synthesis protocols were naturally difficult to reproduce even for senior SAC experts. Protocols containing ambiguous, non-quantifiable language (e.g. “a proper amount”, “heated for some time”) are explicitly excluded to ensure the training data consisted of feasible and actionable instructions. These factors are considered for strict quality control of dataset for fine-tuning. The SAC synthesis protocols from selected papers are classified and labelled by the types of supports (carbon-based, metal oxide and others) and the genre of metal single atoms (block d, block ds, block p and block f), as shown in Fig. 3. Their classification is later applied for stratified sampling to determine the training sets; validation sets and test sets for fine-tuning.

**Fig. 4:**
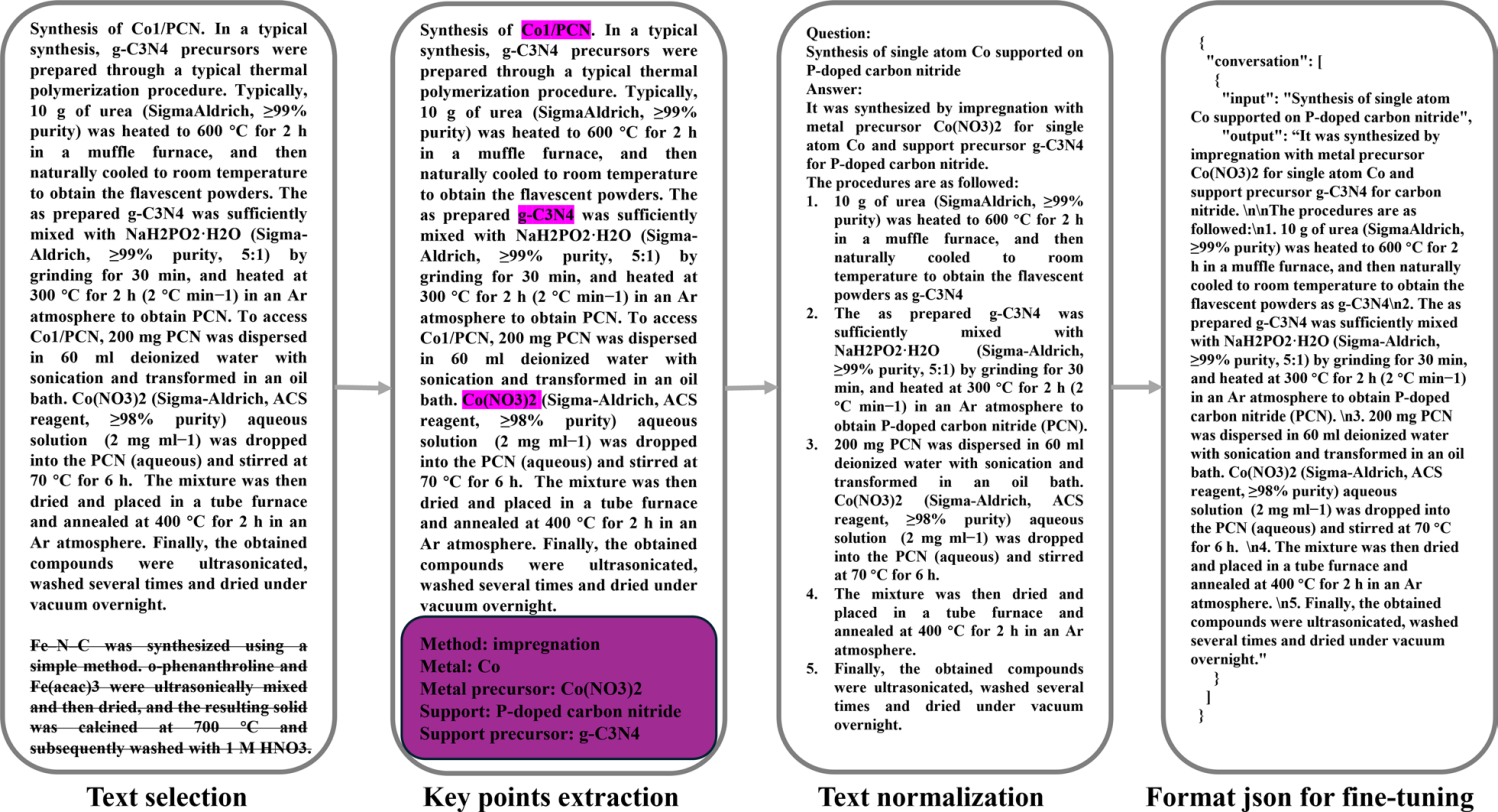
Text from Cao et al. (2019) as an example of text extraction process for fine-tuning [24]

The text extraction process focuses on the experimental sections of these papers, omitting ambiguous narration without important details, and converting unstructured synthesis descriptions into structured text with annotated parameters such as precursors, supports, pyrolysis conditions and post-treatment steps, as shown in Fig. 4. The process is taken manually to ensure the data quality. Data normalization ensures consistency in terminology, resolving variations such as “Fe/NC” into standardized formats like “single atom Fe supported on N-Doped carbon”. This structured dataset enables the model to learn precise synthesis patterns rather than relying on diverse generic text generation. Before the dataset is sent for fine-tuning, it is formatted in JSON to satisfy the requirements of fine-tuning framework applied.

Besides the synthesis procedures, basic knowledge in chemistry and SAC is also included as part of the dataset. This knowledge is supplement for LLMs to potentially understand the principles behind those SAC synthesis procedures. LLMs trained with this knowledge could have the capacity of not only providing the synthesis protocols of a specific SAC but also inferencing the reasons guiding each procedure. Thus, the trained LLMs could potentially optimize SAC synthesis details and predict the synthesis of a new SAC that is never recorded in literature based on this knowledge.

## Results

**Fig. 5:**
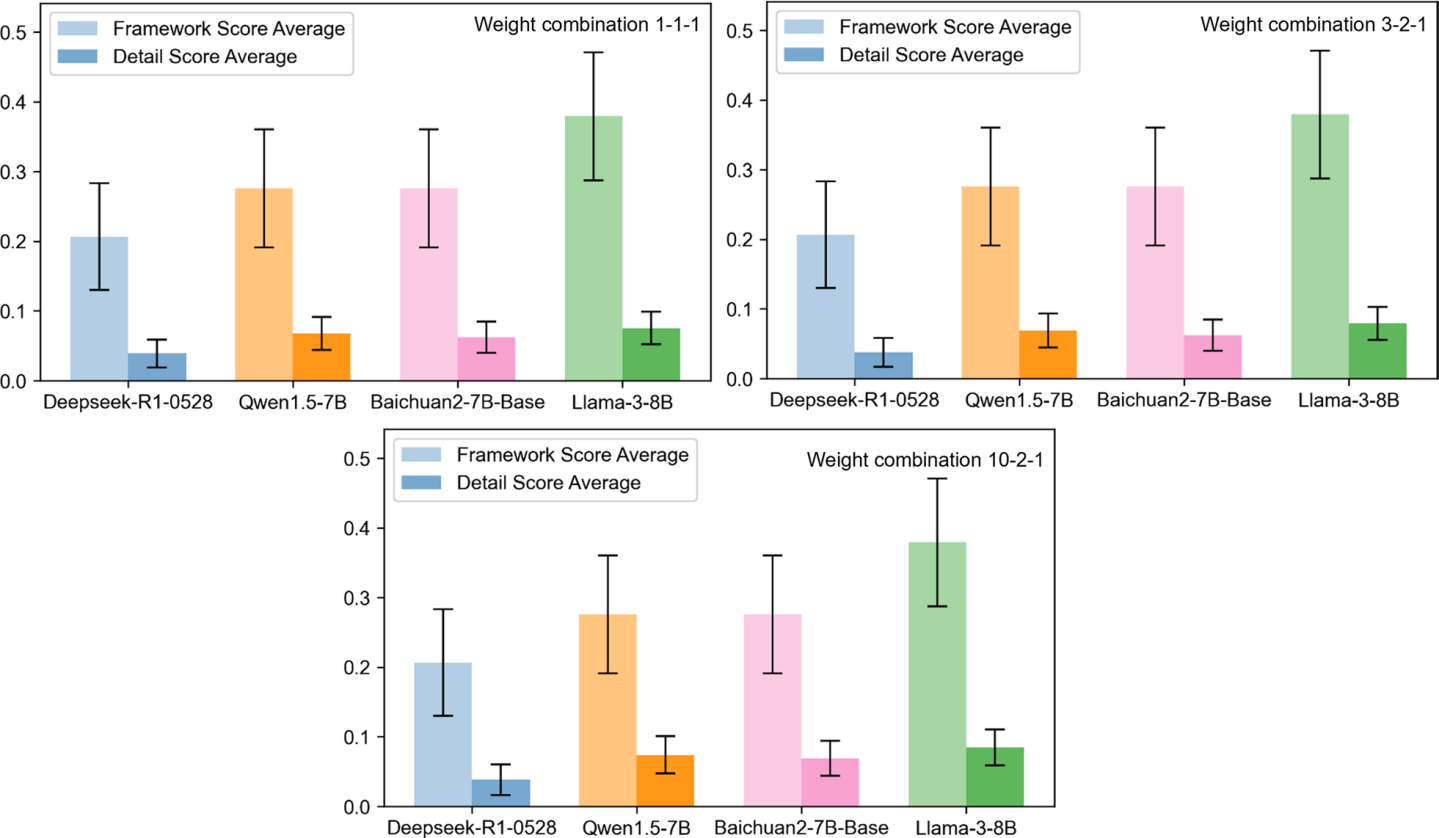
Sensitivity analysis of the evaluation framework. Comparison of SAC expertise metrics of weight combination 1-1-1 (upper left), 3-2-1 (upper right) and 10-2-1 (bottom), demonstrating the robustness of the model rankings regarding weight combination assignment. Scores are averaged over a test set of n = 29 protocols

Crucially, while our fine-tuned model demonstrates a significant capacity for generating chemically plausible frameworks, its average detail score provides a stark, quantitative measure of the profound difficulty in predicting precise experimental parameters. This result does not represent a failure of the model, but rather a key finding of this study: it validates the absolute necessity of a granular, expert-driven validation layer before any AI-generated protocol can be trusted in a physical laboratory. Our framework is the first to decouple and quantify conceptual plausibility from parametric accuracy, providing the essential tool for this critical validation step. The rankings of tested models remain consistent among different weight combinations of the same hierarchy levels. Llama-3-8B shows the highest averaged framework score and averaged detail score and all fine-tuned models significantly outperform commercial Deepseek-R1-0528 by our benchmark (Fig. 5). Meanwhile, untuned Llama-3-8B, untuned Qwen1.5-7B and untuned Baichuan2-7B-Base all yield an averaged framework score of zero and an averaged detail score of zero. This is natural since those models with limited parameters (7B or 8B) probably never learn knowledge from SAC. The actual quantified performance improvements of those fine-tuned models are indeed gain by fine-tunning with curated data but not the models’ original capacity by pretraining.

**Fig. 6:**
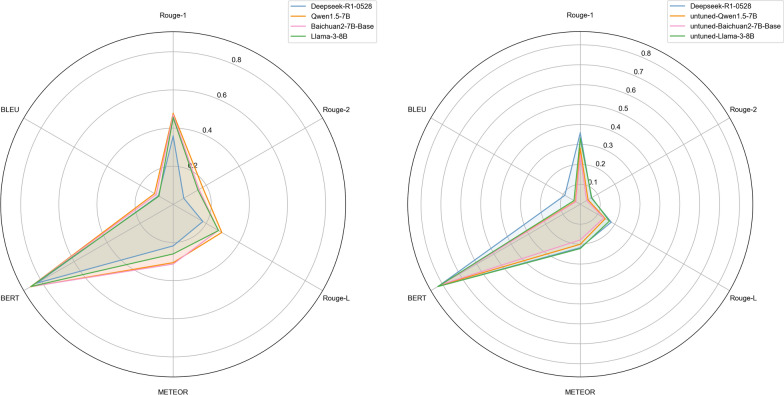
Comparison of NLP metrics among Deepseek-R1-0528 and fine-tuned models (left) and among Deepseek-R1-0528 and untuned models (right). Scores are averaged over a test set of n = 29 protocols

Meanwhile, fined-tuned Qwen1.5-7B and fine-tuned Baichuan2-7B-Base show slightly better capacity in language processing than fined-tuned Llama-3-8B by all NLP metrics, as shown on the left of Fig. 6. On the contrary, Llama-3-8B shows the best performance by SAC expertise metrics. The fine-tuned models were trained on data that underwent terminology normalization and structural formatting, so their outputs would naturally align more closely with the reference text (prediction target). As a result, the lowest NLP scores of the Deepseek-R1-0528 may partly reflect format mismatch. On the contrary, Deepseek-R1-0528 outperformed all untuned models in language processing as shown on the right of Fig. 6, since it is considered one of the most competitive LLMs and it has 671B parameters, which is significantly larger than those of other untuned models (7B or 8B).

The performance differences among LLMs after fine-tuning with the same parameters trace mostly to the differences of pretraining. Llama-3-8B was pretrained on ~ 15 trillion tokens with emphasis on data quality, careful deduplication/filtering, annealing small high-quality math/code subsets and scaling-law driven design [25]. In particular, it continued to be pretrained by extended-context up to 128 k tokens. Baichuan2-7B-Base was pretrained from scratch on ~ 2.6 trillion tokens, focusing on massive token exposure even for relatively small parameter counts, strong deduplication/clustering and data scoring, tokenizer engineering, careful scaling-law fits and many intermediate checkpoints released to inspect training dynamics [26]. Qwen1.5-7B was pretrained on ~ 2.4 trillion tokens with multilingual, code and instruction-style data [27]. It reports careful tokenization and pretraining alignment pipelines.

**Fig. 7:**
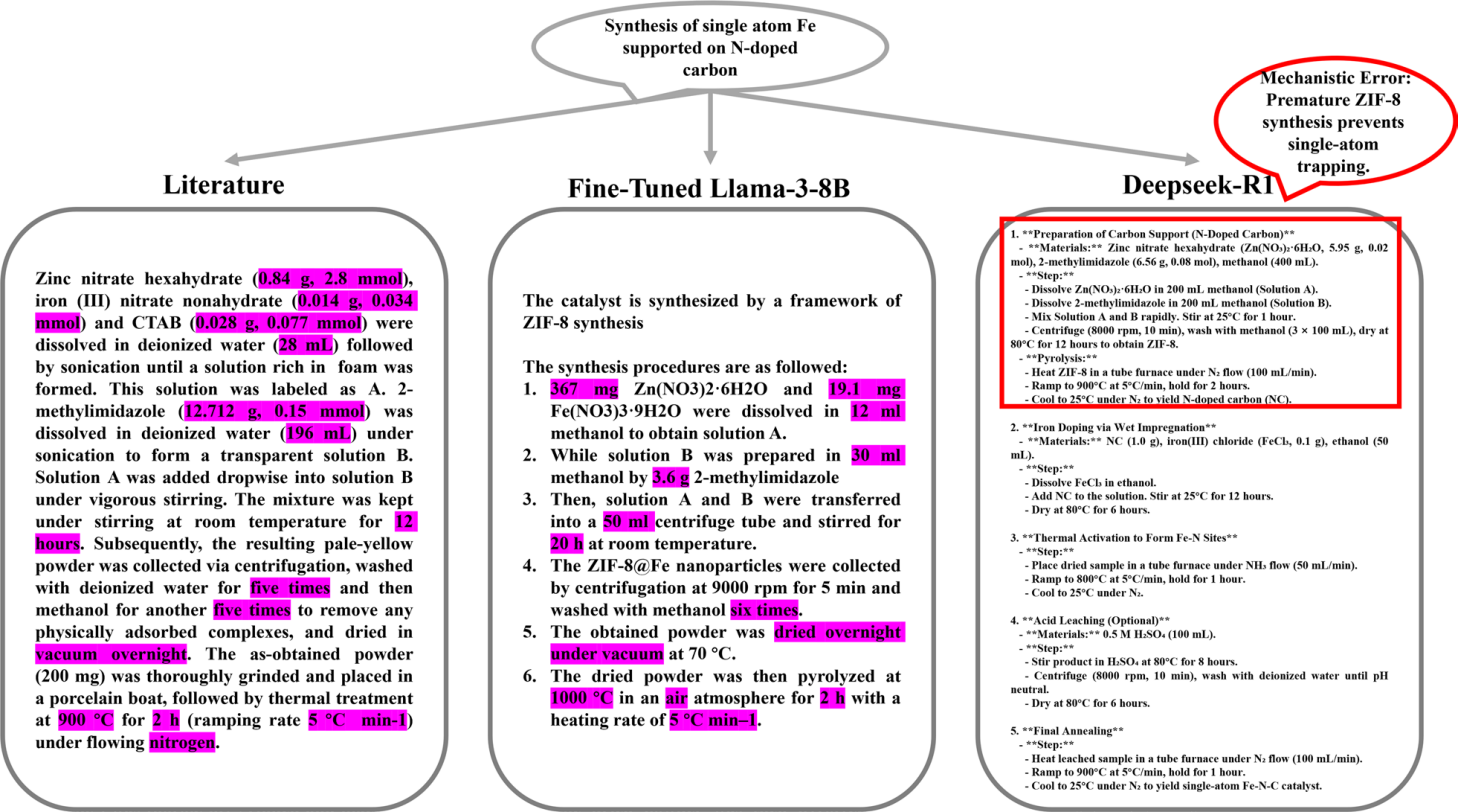
Fe-N-C synthesis protocol generated by literature [28], fine-tuned Llama-3-8B and Deepseek-R1-0528. Please note that the proposed scheme of Deepseek-R1-0528 (right) contains a fundamental mechanistic error (i.e., the initial synthesis of ZIF-8), which represents a typical case of “scientific illusion”. This stands in sharp contrast to the chemically plausible process generated by Llama-3-8B (middle)

In the case of synthesizing single atom Fe supported on N-doped carbon (Fe-N-C) via wet impregnation as shown in Fig. 7, the fine-tuned Llama-3-8B model correctly provided precursor, support and major steps, whereas the commercial Deepseek-R1-0528 model incorrectly suggested to synthesize ZIF-8 first, then impregnate Fe salts with prepared ZIF-8 and activate by ammonia. ZIF-8 is a type of metal–organic framework (MOF) commonly used as a template to load single atoms for SAC synthesis. This approach is highly likely to result in the formation of Fe nanoparticles on the exterior of the MOF crystals, rather than isolated atoms embedded within the N-doped carbon matrix derived from ZIF-8 ligand pyrolysis. This indicates that Deepseek-R1-0528 embodies a fundamental misinterpretation of the pyrolysis-driven atomic trapping mechanism, a cornerstone principle for this class of MOF-derived SAC synthesis. Furthermore, our fine-tuned model Llama-3-8B generated quantitative values for precursor amounts that, while not exact, were in the correct order of magnitude (e.g., 367 mg vs. 840 mg), demonstrating a superior grasp of the synthesis recipe compared to the commercial model.

## Discussion

As an initial benchmark in this field, our adoption of the 3-2-1 weighting scheme is based on widely accepted physicochemical principles in SAC synthesis (e.g., calcination temperature determines single-atom anchoring and is far more critical than washing time). Furthermore, through sensitivity analyses with different weight combinations (Fig. 5), we demonstrate that even with some subjectivity in the weights, the relative ranking conclusions regarding model performance remain robust.

Our results suggest that the most effective near-term role for large language models (LLMs) in materials synthesis is not as fully autonomous designers, but as highly knowledgeable digital apprentices. These models can efficiently overcome the “cold start” problem by generating plausible first-draft synthesis protocols, thus liberating human experts from exhaustive literature searches and repetitive formulation tasks. In this human-AI collaborative paradigm, our validation framework functions as the essential supervisory tool, allowing expert chemists to rigorously evaluate, refine, and ultimately trust the model’s proposals before allocating valuable laboratory resources.

Beyond demonstrating the ability to generate high-fidelity synthesis protocols, our study establishes a new methodological benchmark for evaluating generative LLMs in chemistry. The failure of strict NLP-based metrics arises from a mismatch between linguistic assumptions and the physical nature of chemical synthesis. Chemical reactions evolve on constrained energy landscapes, where a limited number of parameters, such as atomic coordination and atom-trapping sites, disproportionately determine reaction pathways and outcomes. Small perturbations to these variables can induce non-linear changes in kinetics and stability, whereas variations in textual expression are physically irrelevant. Strict NLP metrics therefore overemphasize superficial differences while neglecting mechanistically critical factors. In contrast, our benchmark encodes the hierarchical influence of synthesis parameters according to their impact on reaction energetics and electronic structure. These weight combinations reflect objective physicochemical mechanisms rather than subjective preference, enabling chemically meaningful comparisons that align with the causal determinants of reaction behavior.

Our findings also offer concrete guidance for future model development. The superior reasoning performance observed in models pre-trained on structured data sources such as code and mathematics, indicates that domain-specific alignment, built upon foundational reasoning ability, plays a crucial role in scientific competence. This insight highlights the importance of developing future foundation models for science with architectures and training data explicitly designed to support structured reasoning, quantitative consistency, and logical generalization.

Our validation framework directly addresses a critical governance gap in emerging AI agent platforms like ChemCrow [29]. However, these systems typically lack an internal mechanism for assessing the scientific validity of their self-generated action plans prior to execution, as shown in Table 3. Our proposed validation framework fills this critical gap by serving as an internal verification module within the agent’s decision-making loop. By enabling rapid and quantitative risk assessment of generated protocols before resource-intensive physical experiments or computational simulations are initiated, this framework introduces a necessary layer of self-regulation. In doing so, it addresses a central bottleneck in the evolution of truly autonomous scientific agents, transforming AI from a mere “tool orchestrator” into a self-reflective scientific reasoning engine capable of quality control and rational judgment.

**Table 3:** Positioning the validation framework within the AI-driven scientific workflow

Tool/method category	Primary role in DBTL cycle	Core AI mission	Key limitation addressed by this work
This work (validation framework)	Validate (design) and learn	Assess scientific soundness of generated protocols	Addresses the "trust bottleneck" and lack of domain-specific reliability metrics for generative models
Retrosynthesis planners (e.g., Chemma)	Design (propose route)	Predict reaction pathways for known molecules	Generates pathways, but does not validate or provide precise, reproducible experimental parameters
Property predictors (e.g., AlphaFold)	Design (screen candidates)	Predict structure/properties from sequence/formula	Predicts a final property, but does not generate or validate the synthesis *process* to create it
AI synthesis agents (e.g., ChemCrow)	Design and orchestrate (Build)	Execute tasks using a suite of tools	Relies on external tools; this framework provides the missing *internal validation module* to assess its own generated plans

The framework’s reliance on intensive manual effort for data curation and evaluation hinders its scalability to other materials or domains. Future work should employ high-quality LLM-based agents to automate this process while preserving the chemically aware evaluation philosophy. However, a key limitation remains: because scores reflect agreement with established literature, a novel yet valid synthesis route would score poorly. Thus, the benchmark primarily rewards reproducing known procedures rather than genuine scientific reasoning or discovery. Overcoming this is essential to advancing autonomous scientific agents from simple tool orchestrators into self-reflective engines capable of quality control and rational judgment.

## Conclusion

This study introduces and validates a quantitative framework for assessing the scientific reliability of synthesis plans generated by LLMs. The successful fine-tuning of LLMs for generating detailed SAC synthesis protocols marks a significant advancement in SAC synthesis. We build a multi-dimensional assessment system with newly-defined metrics focused on SAC expertise, which validates the accuracy of LLMs-produced outputs. Comparison between fine-tuned LLMs reveals the significance of pretraining on downstream scientific tasks. It provides useful guides to select LLMs for SAC-related research among numerous open-source LLMs nowadays. The proposed tool addresses a critical validation bottleneck in automated materials discovery and represents a necessary step toward deploying trustworthy AI agents in self-driving laboratories.

## Supplementary Information

Additional file 1.

## Data Availability

The code and data for this study are openly available at [https://github.com/aobozhang2025/data-driven-LLM-SAC] (https://github.com/aobozhang2025/data-driven-LLM-SAC). Access to Deepseek-R1-0528 is commercially available at [https://www.modelscope.cn/models/deepseek-ai/DeepSeek-R1-0528/summary] (https://www.modelscope.cn/models/deepseek-ai/DeepSeek-R1-0528/summary).
