# Supplementary material for: A chemically-aware validation framework for benchmarking large language models in materials synthesis planning
Source: J Cheminform. 2026 May 24;18:100. doi: 10.1186/s13321-026-01222-5 (PMC13383416; doi:10.1186/s13321-026-01222-5)
Supplement: Supplementary file 1 — Additional file 1. [file 13321_2026_1222_MOESM1_ESM.docx]

**Supporting Information**

A Chemically-Aware Validation Framework for Benchmarking Large Language Models in Materials Synthesis Planning

Aobo Zhang^1^*

^1^Department of Chemistry, Tsinghua University, Beijing 100084, China.

**Table of Contents**

Data Preparation page 2-3

Hardware Setup page 4

Software Setup page 4

Fine-Tune Details page 5

Evaluation Details page 6-7

Prompt Robust Test page 8

Numerical Value for Fig.5 page 9

References page 10

**Data Preparation**

The papers selected were searched from the journals official websites such as <https://www.nature.com> and <https://pubs.acs.org/journal/jacsat> with keywords related to single-atom catalysis (SAC). The metadata of chosen papers were saved by literature management tool, Zotero. Zotero automatically saved papers contents and supporting information as pdfs locally. The synthesis descriptions were manually selected, cleaned and saved in txt files named by the DOIs of papers. The information of each SAC synthesis protocol was recorded on an excel file for future references and management.

The part of SAC synthesis from each paper are manually copy-paste to a txt file with each file named by the DOI of the paper and the number of SAC in the paper. Then the manual data curation will first omit synthesis protocols with ambiguous narration (like “for some amount”) and/or without important details. Then manually summarize the important details including single atom metal site, metal precursor, support, support precursor if any and synthesis method (impregnation for example). The question would be of the format “synthesis of single atom X supported on S”. The answer would be of the format that the first sentence is a summary of the synthesis protocol with all important information, such as “it was synthesized by impregnation with metal precursor XX for single atom X and support precursor SS for support S”. Then with a new paragraph with sentence “the procedures are as followed:”. Then narrate the steps with its sequence numbers at beginning in a new paragraph for each step.

Common mapping rules to standardize synthesis description are listed in the table below

| X/NC | Single atom X supported on N-doped carbon, denoted by X/NC |
| --- | --- |
| Argon atmosphere | Ar atmosphere |
| 6 hours | 6 h |
| iron(III) nitrate nonahydrate | Fe(NO3)3·9H2O |
| calcined at 900 C | 900 °C |
| five times | 5 times |

The ready-to-use synthesis protocols in txt files were converted to question-answer pairs in JSON format by a Python script, which could be easily generated by commercial LLMs. The JSON files were later applied for fine-tunning. The JSON file is of the format:

{

“conversation”: [

{

“input”: “{question}”

“output”: “{answer}”

}

]

}

The background knowledge in SAC was collected from textbooks, related answers from online recourses such as Wikipedia. This knowledge was double-checked by senior experts in SAC for many years. It was also summarized in question-answer pairs in JSON files for fine-tunning. The background knowledge included common SAC synthesis procedures such as calcination, chemical properties of metal atoms and their precursors in SAC and chemical properties of supports in SAC.


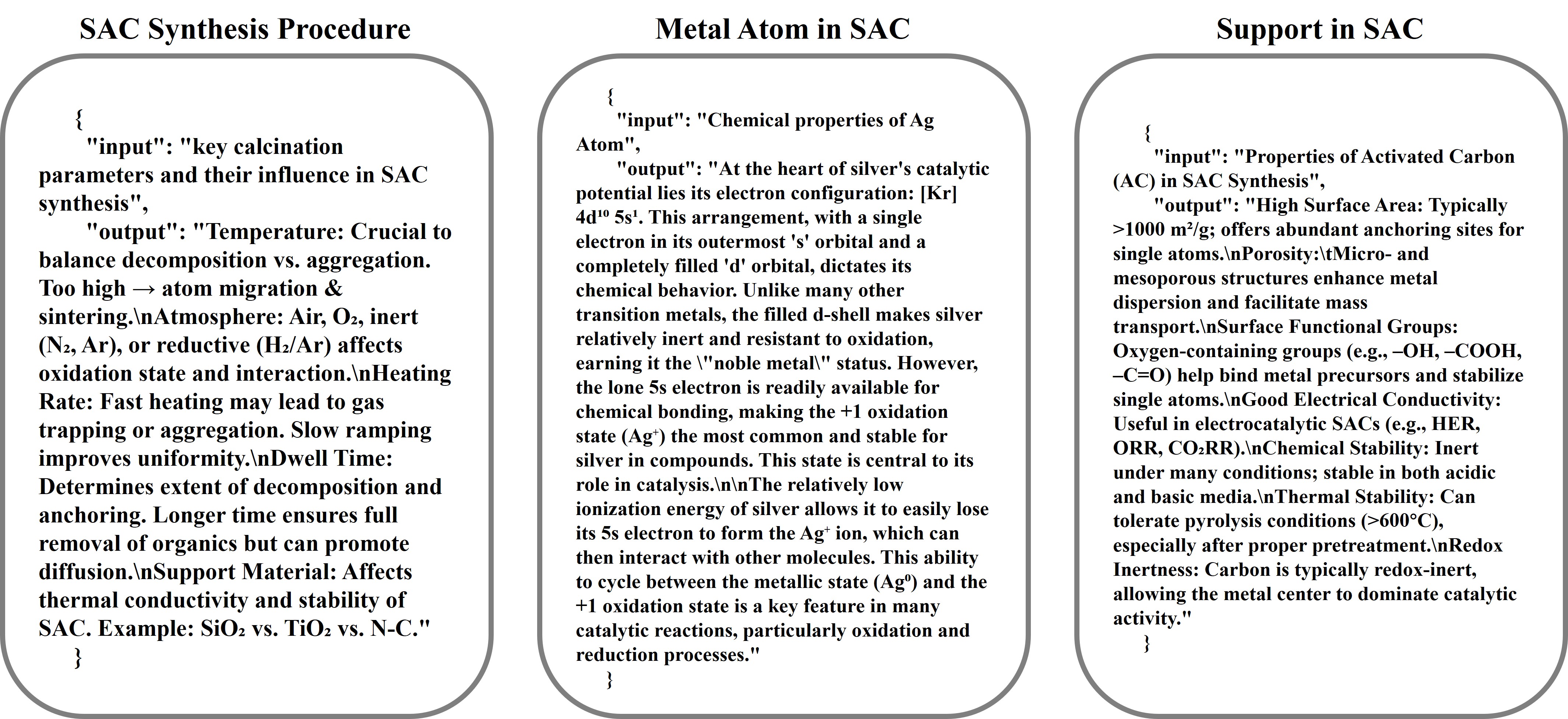


**Figure S1.** Examples of background knowledge question-answer pairs in SAC synthesis procedures, metal atoms in SAC and support in SAC.

**Hardware Setup**

The computing server was composed of two Intel Sapphire Rapids 8462Y+ [32C@2.8GHz](mailto:32C@2.8GHz) CPU with a memory of 2048 GB and eight NVIDIA Tesla H800 SXM5 GPU with a graphics memory of 8*80 GB. The server was installed Ubuntu 22.04 operating system. CUDA 12.6 was loaded for computation with NVIDIA graphic cards.

**Software Setup**

The open-source LLMs for fine-tunning were downloaded from <https://www.modelscope.cn> to the computing server locally by SDK method.

The fine-tune framework Xtuner of version v0.2.0rc0 was installed by the instructions from its official website on github <https://github.com/InternLM/xtuner>. Xtuner is an efficient, flexible and full-featured toolkit for fine-tuning large models. It supports fine-tuning in various algorithms such as QLoRA, LoRA and full parameter fine-tune for mainstream open-source LLMs^1^. It is based on mainstream Python modules for LLMs and fine-tuning such as Transformers 4.48.0 and PyTorch 2.5.1. The version of all Python module dependencies can be found on Xtuner official website. The fine-tuning was setup by a configure file in Python provided by Xtuner with all the fine-tune hyperparameters to be determined.

**Fine-Tune details**

The fine-tune algorithms applied in this paper was Quantized Low Rank Adaptation (QLoRA). QLoRA was explained in the method section of this paper. All hyperparameters remained unchanged from default except some key hyperparameters that significantly affected the efficiency of fine-tunning. Multiple combinations of hyperparameters were tried to speed up fine-tuning, maximize the efficiency of fine-tuning and avoid overfitting or gradient explosion. The final optimized hyperparameter combination was determined by the LLMs, the size of dataset and the hardware.

The key hyperparameters were finally determined as shown in Supplementary Table 1.

| Hyperparameter Name | Explanation | Value |
| --- | --- | --- |
| Prompt_template | Same for all LLMs | default |
| System_template | Same for all LLMs | alpaca |
| Max_length | Max tokens for output | 2048 |
| Batch_size | Sample size for each turn | 50 |
| Max_epochs | Max epoch | 400 |
| Optim_type | Learning rate optimization method | AdamW |
| lr | Initial learning rate | 5e-5 |
| Save_steps | Save weights every number of steps | 200 |
| load_in_4bit | Quantized in 4 bits | True |
| r | The rank for adaptation | 64 |
| Lora_alpha | Usually 1 or 2 times r by mainstream experience | 128 |

**Table S1.** Values of Significant hyperparameters applied for all fine-tuned models.

**Evaluation details**

The framework score and detail score were recorded as explained in the methods section of this paper. Here we provide detailed examples to further illustrate the calculation of these scores with the following figure.


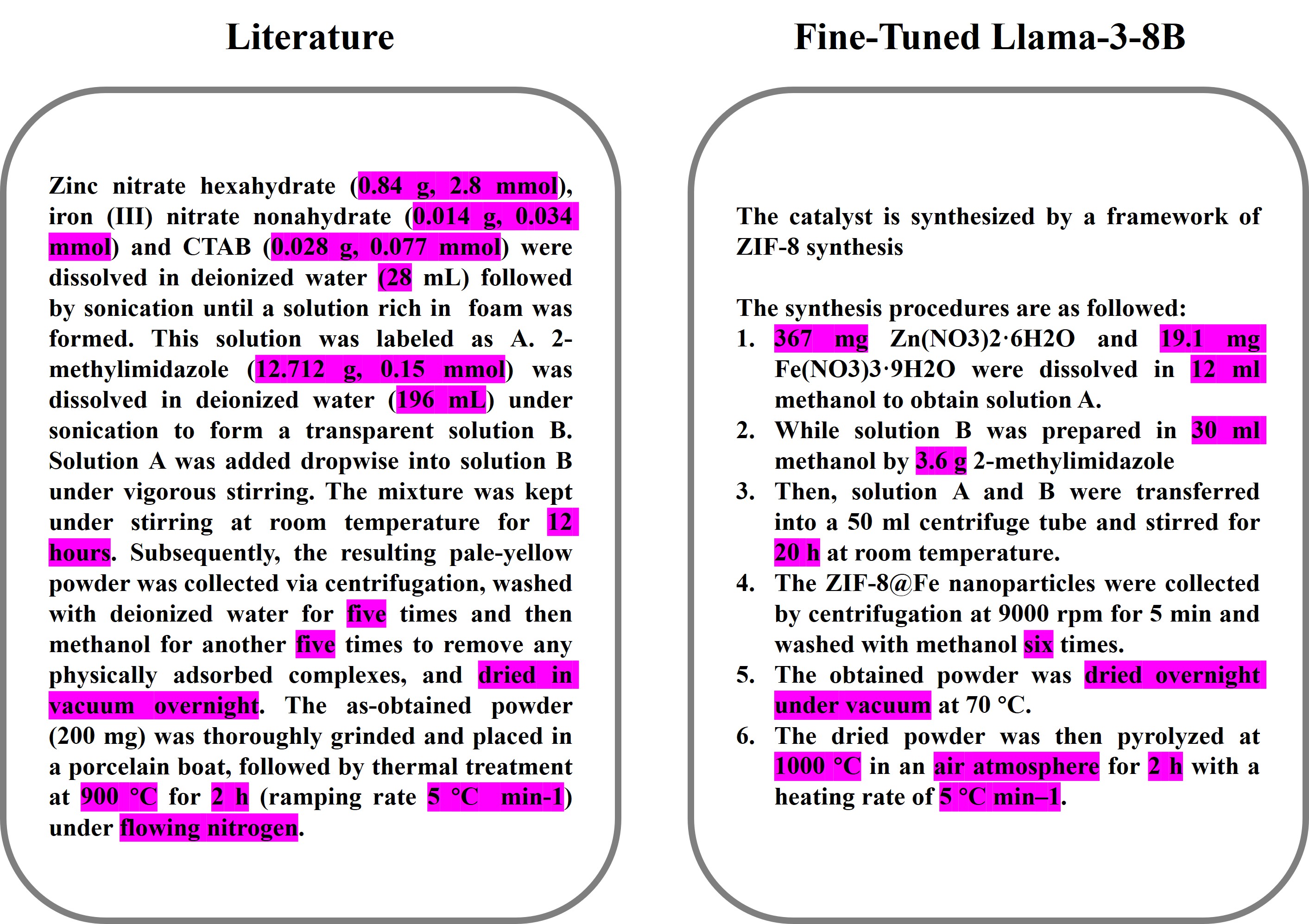


**Figure S2**. Comparison of Fe-N-C synthesis designs between from literature^2^ and from fine-tuned Llama-3-8B.

The framework of Fe-N-C synthesis generated by fine-tuned Llama-3-8B roughly matched the synthesis descriptions from literature, which was a complete synthesis design based on the synthesis of ZIF-8 framework^2^. The overall procedures matched very well including impregnation, cleaning, drying, and calcination. The precursor and support selected, together with other related chemicals, were correct. Thus, the framework score of this synthesis design was “pass”.

The score of each detail was first classified by three levels of hierarchy. We applied weight combination 3-2-1 for instance. The top hierarchy level with weight 3 included factors: calcination temperature, calcination atmosphere, calcination time. The second hierarchy level with weight 2 included factors: chemical weight, ramping time. The least hierarchy level with weight 1 included any other factors such as drying time, centrifugation times, etc.

There were 14 important details of this Fe-N-C synthesis as marked in pink in Figure S2. Each detail was weighted as explained by three levels of importance. The total weight summed was 27. For each numerical detail such as the weight of zinc nitrate hexahydrate, it was 0.84 g in literature while 367 mg generated by Baichuan-7B-Base. It was the amount of chemical so weighted by 2. The score for this specific detail was calculated as 2/27 * (1 - |0.367 – 0.84| / 0.84) = 0.0324. The weight of CTAB was missing in the generated synthesis so the score of this detail was 0. For each non-numerical detail such as “dried in vacuum overnight” from literature, the score of this detail was 0 if it was missing or stated incorrectly. In this case, the score of this detail was a full score of 1/27 since it was mentioned correctly as “dried overnight under vacuum”.

After the scores of all details were determined, the final detail score was the sum of all those scores. In this case, the final detail score of this Fe-N-C synthesis was calculated as 0.5064 out of 1. The calculations of all scores of details were listed in Table 2 below.

| Literature Detail | Generated Detail | Weight | Calculation | Final Score |
| --- | --- | --- | --- | --- |
| 0.84 g | 367 mg | 2 | 2/27 * (1 - \|0.367 – 0.84\| / 0.84) | 0.0324 |
| 0.014 g | 19.1 mg | 2 | 2/27 * (1 – \|0.0191 – 0.014\| / 0.014) | 0.0471 |
| 0.028 g |  | 2 | 0 | 0 |
| 28 mL | 12 mL | 2 | 2/27 * (1 - \|12 - 28\| / 28) | 0.0317 |
| 12.712 g | 3.6 g | 2 | 2/27 * (1- \|3.6 – 12.712\| / 12.712) | 0.0210 |
| 196 mL | 30 mL | 2 | 2/27 * (1 - \|30 - 196\| / 196) | 0.0113 |
| 12 hours | 20 h | 1 | 1/27 * (1 – \|20 - 12\| / 12) | 0.0123 |
| Five times |  | 1 | 0 | 0 |
| Five times | Six times | 1 | 1/27 * (1 – \|6 - 5\| / 5) | 0.0296 |
| Dried in vacuum overnight | Dried overnight under vacuum | 1 | 1/27 | 0.0370 |
| 900 C | 1000 C | 3 | 3/27 * (1 – \|1000 - 900\| / 1000) | 0.0987 |
| 2 h | 2 h | 3 | 3/27 | 0.1111 |
| 5 C / min | 5 C / min | 2 | 2/27 | 0.0741 |
| Flowing nitrogen | Air atmosphere | 3 | 0 | 0 |

**Table S2**. Details and their score calculations of this specific Fe-N-C synthesis case.

Note: The above example, in which the model generated a valid framework, is provided to clearly illustrate the calculation methodology for the detail score. This case represents one of the model's stronger performances, and the calculated score of 0.5064 should not be mistaken for the model's average performance across the entire test set (0.0792), as reported in the main manuscript.

**Prompt Robustness Test**

The model tested is fine-tuned Llama-3-8B and the weight 1-2-3 is applied.

Below is the prompt robust test for prompt “synthesis of single atom Fe supported on N-doped carbon”, “synthesis of Fe-N-C”, “synthesis of Fe/NC”

| Prompt | Fe supported on N-doped carbon | Fe-N-C | Fe/NC |
| --- | --- | --- | --- |
| Framework score | 1 | 1 | 1 |
| Detail score | 0.5064 | 0.3953 | 0.4694 |

The framework score is with standard deviation 0 and the detail score is with standard deviation 0.04619.

Below is another prompt robust test for prompt “preparation of Co supported on N-doped Carbon”, “preparation of Co-N-C”, “preparation of Co/NC”

| Prompt | Co supported on N-doped carbon | Co-N-C | Co/NC |
| --- | --- | --- | --- |
| Framework score | 1 | 1 | 1 |
| Detail score | 0.2578 | 0.2638 | 0.2522 |

The framework score is with standard deviation 0 and the detail score is with standard deviation 0.04736.

Below is another prompt robust test for prompt “preparation of Ni supported on N-doped Carbon”, “preparation of Ni-N-C”, “preparation of Ni/NC”

| Prompt | Ni supported on N-doped carbon | Ni-N-C | Ni/NC |
| --- | --- | --- | --- |
| Framework score | 1 | 1 | 1 |
| Detail score | 0.1176 | 0.1209 | 0.0862 |

The framework score is with standard deviation 0 and the detail score is with standard deviation 0.01564.

**Numerical Value for Fig.5**

For weight combination 1-1-1

| Model | Deepseek-R1-0528 | Qwen1.5-7B | Baichuan2-7B-Base | Llama-3-8B |
| --- | --- | --- | --- | --- |
| Framework score | 0.2069 ± 0.0766 | 0.2759 ± 0.0845 | 0.2759 ± 0.0845 | 0.3793 ± 0.0917 |
| Detail score | 0.0393 ± 0.0200 | 0.0679 ± 0.0236 | 0.0623 ± 0.0223 | 0.0755 ± 0.0233 |

For weight combination 1-2-3

| Model | Deepseek-R1-0528 | Qwen1.5-7B | Baichuan2-7B-Base | Llama-3-8B |
| --- | --- | --- | --- | --- |
| Framework score | 0.2069 ± 0.0766 | 0.2759 ± 0.0845 | 0.2759 ± 0.0845 | 0.3793 ± 0.0917 |
| Detail score | 0.0377 ± 0.0206 | 0.0692 ± 0.0243 | 0.0624 ± 0.0223 | 0.0792 ± 0.0239 |

For weight combination 1-2-10

| Model | Deepseek-R1-0528 | Qwen1.5-7B | Baichuan2-7B-Base | Llama-3-8B |
| --- | --- | --- | --- | --- |
| Framework score | 0.2069 ± 0.0766 | 0.2759 ± 0.0845 | 0.2759 ± 0.0845 | 0.3793 ± 0.0917 |
| Detail score | 0.0384 ± 0.0220 | 0.0742 ± 0.0266 | 0.0692 ± 0.0251 | 0.0848 ± 0.0255 |

**References**

(1) XTuner Contributors. XTuner: A Toolkit for Efficiently Fine-Tuning LLM, https://github.com/InternLM/xtuner (accessed 9 August 2025)

(2) Zhang, Y.-X.; Zhang, S.; Huang, H.; Liu, X.; Li, B.; Lee, Y.; Wang, X.; Bai, Y.; Sun, M.; Wu, Y.; Gong, S.; Liu, X.; Zhuang, Z.; Tan, T.; Niu, Z. General Synthesis of a Diatomic Catalyst Library via a Macrocyclic Precursor-Mediated Approach. *J. Am. Chem. Soc.* 2023, *145* (8), 4819–4827.
